# Anti-Cancer Agents in Proliferation and Cell Death: The Calcium Connection

**DOI:** 10.3390/ijms20123017

**Published:** 2019-06-20

**Authors:** Elizabeth Varghese, Samson Mathews Samuel, Zuhair Sadiq, Peter Kubatka, Alena Liskova, Jozef Benacka, Peter Pazinka, Peter Kruzliak, Dietrich Büsselberg

**Affiliations:** 1 Department of Physiology and Biophysics, Weill Cornell Medicine-Qatar, Education City, Qatar Foundation, Doha P.O. Box 24144, Qatar; elv2007@qatar-med.cornell.edu (E.V.); sms2016@qatar-med.cornell.edu (S.M.S.); zuhairsadiq2018@u.northwestern.edu (Z.S.); 2 Department of Medical Biology and Department of Experimental Carcinogenesis, Division of Oncology, Biomedical Center Martin, Jessenius Faculty of Medicine, Comenius University in Bratislava, 036 01 Martin, Slovakia; kubatka@jfmed.uniba.sk; 3 Department of Obstetrics and Gynecology, Jessenius Faculty of Medicine, Comenius University in Bratislava, 036 01 Martin, Slovakia; alenka.liskova@gmail.com; 4 Faculty Health and Social Work, Trnava University, 918 43 Trnava, Slovakia; jozef.benacka@centrum.cz; 5 Department of Surgery, Faculty of Medicine, Pavol Jozef Safarik University and Louise Pasteur University Hospital, 04022 Kosice, Slovakia; peter.pazinka@seznam.cz; 6 Department of Internal Medicine, Brothers of Mercy Hospital, Polni 553/3, 63900 Brno, Czech Republic; peter.kruzliak@savba.sk; 7 2nd Department of Surgery, Faculty of Medicine, Masaryk University and St. Anne’s University Hospital, 65692 Brno, Czech Republic

**Keywords:** Intracellular calcium, anti-cancer drugs, apoptosis, proliferation

## Abstract

Calcium (Ca^2+^) signaling and the modulation of intracellular calcium ([Ca^2+^]_i_) levels play critical roles in several key processes that regulate cellular survival, growth, differentiation, metabolism, and death in normal cells. On the other hand, aberrant Ca^2+^-signaling and loss of [Ca^2+^]_i_ homeostasis contributes to tumor initiation proliferation, angiogenesis, and other key processes that support tumor progression in several different cancers. Currently, chemically and functionally distinct drugs are used as chemotherapeutic agents in the treatment and management of cancer among which certain anti-cancer drugs reportedly suppress pro-survival signals and activate pro-apoptotic signaling through modulation of Ca^2+^-signaling-dependent mechanisms. Most importantly, the modulation of [Ca^2+^]_i_ levels via the endoplasmic reticulum-mitochondrial axis and corresponding action of channels and pumps within the plasma membrane play an important role in the survival and death of cancer cells. The endoplasmic reticulum-mitochondrial axis is of prime importance when considering Ca^2+^-signaling-dependent anti-cancer drug targets. This review discusses how calcium signaling is targeted by anti-cancer drugs and highlights the role of calcium signaling in epigenetic modification and the Warburg effect in tumorigenesis.

## 1. Intracellular Calcium Homeostasis and Calcium Signaling 

Intracellular calcium ([Ca^2+^]_i_) is an important second messenger involved in cellular functions of muscles, neurons, immune cells, oocytes and others, modulating enzyme secretion, gene activation, proliferation, apoptosis, cell cycle progression, fertilization, and release of neurotransmitters [1,2,3]. Aberrant [Ca^2+^]_i_-signaling has been implicated in diseases such as Alzheimer’s, cancer, congestive heart failure, and diabetes [4,5,6]. In cancer, [Ca^2+^]_i_-signaling is involved in various processes of tumorigenesis such as proliferation, migration, angiogenesis, and evasion of apoptosis [7,8]. 

**Figure 1:**
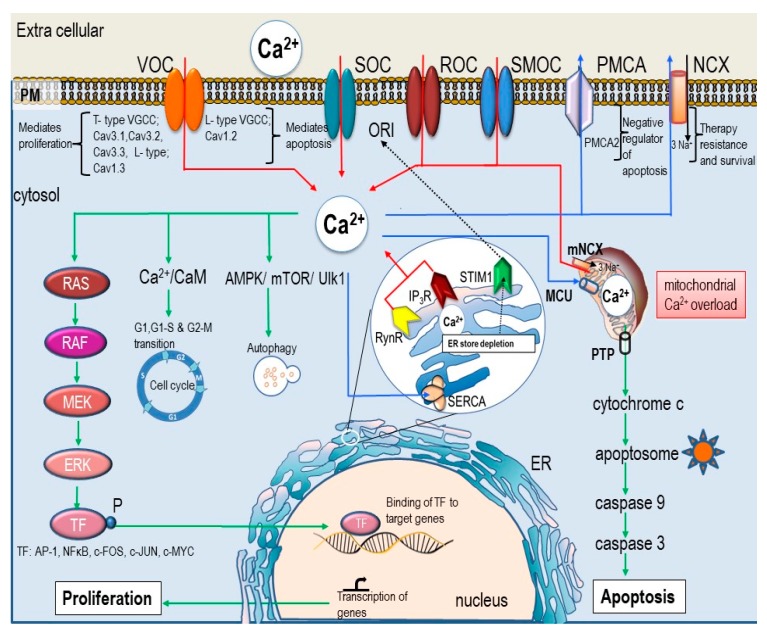
Ca^2+^-signaling in proliferation and apoptosis. [Ca^2+^]_i_ homeostasis of the cell is strictly regulated by various channels and pumps on plasma membrane, ER, and mitochondria. Intracellular calcium flux is indicated by arrows where red arrows indicate influx of Ca^2+^ ions, blue arrows indicate Ca^2+^ efflux and sequestration of Ca^2+^ into the stores, and green arrows indicate cell signaling pathways which are activated by [Ca^2+^]_i._ Primarily Ca^2+^ influx from the extra-cellular space is controlled by VOC (voltage operated calcium channel), SOC (store-operated Orai channels), SMOC (second messenger-operated channel), or ROC (receptor-operated channel), and Ca^2+^ efflux is mediated via (NCX (Na^+^/Ca^2+^ exchanger) and PMCA (plasma membrane Ca^2+^-ATPase). Ca^2+^ release from the ER stores is facilitated via RyR (ryanodine receptors) and IP_3_R (IP_3_ receptor). Uptake of calcium from the cytosol is an energy driven process mediated by SERCA (sarcoplasmic/ER Ca^2+^-ATPase). Additionally, mitochondria and associated proteins (including mitochondrial calcium uniporter (MCU), voltage dependent anion channel (VDAC), and mitochondrial Na^+^/Ca^2+^ exchanger (mNCX) are also related to [Ca^2+^]_i_-regulation. Mitochondrial-Ca^2+^ controls ATP synthesis, apoptosis, ROS generation, and biosynthesis and can determine the fate of the cell. The concentration of Ca^2+^ in the cytosol is maintained at a low level (100 nM) in comparison to the extra cellular Ca^2+^ (1mM). Any change in the [Ca^2+^]_i_ results in signal transduction initiating various cellular process such as apoptosis, proliferation, and cell division. The type of signal depends on the duration, amplitude, localization, frequency, and oscillation. Sustained high level of Ca^2+^ in the mitochondria causes the release of cytochrome *c* and subsequently triggers death signals via caspase activation. More Ca^2+^ influx from the ion channels on the plasma membrane can trigger either proliferation (via T-type) or apoptosis (via L-type). Increased Ca^2+^ entry through the SOC channel promotes proliferation [32].

The concentration of Ca^2+^ in different compartments of a cell differs greatly ([Ca^2+^]_i_: 100 nM; extracellular calcium [Ca^2+^]_o_: 1–1.5mM; in the endoplasmic reticulum (ER): 0.5–1 mM; in the mitochondria: 100–200 nM (but can accumulate 10–20 fold upon activation) [9]. [Ca^2+^]_i_ is tightly maintained at a low concentration by numerous regulating mechanisms, that include both active and passive mechanisms, e.g., channels or pumps on the plasma membrane, ER, mitochondria, and cytosol (Figure 1) [10,11,12,13]. In addition to channels and pumps, [Ca^2+^]_i_ is modulated by a heterogeneous group of calcium binding proteins (CBP), including S100 proteins, calmodulin, and calcineurin. Binding of Ca^2+^ with CBP regulates signal transduction and gene expression [14]. Undoubtedly, the nucleus acts as a major compartment in which Ca^2+^ is sequestered. Nuclear Ca^2+^ has specific biological functions involved in gene expression and regulation [15]. The involvement of the nucleus in [Ca^2+^]_i_-regulation and its role in cell proliferation is extensively reviewed by Resende and colleagues [16]. Importantly, some studies proposed nuclear calcium signaling as an independent entity producing localized calcium-signaling and triggering the transcription of genes related to cell proliferation [17]. On the contrary, it is reported that nuclear [Ca^2+^] is dependent on changes in [Ca^2+^]_i_ [18,19].

[Ca^2+^]_i_-signaling is initiated by the entry of Ca^2+^ from an extracellular pool or by releasing Ca^2+^ from ER stores or mitochondria. This increases [Ca^2+^]_i_ from 100 nM (at rest) to approximately 1000 nM generating an “ON” signal for multiple processes. As a prolonged increase in [Ca^2+^]_i_ may be harmful, the [Ca^2+^]_i_ signals are spatially and temporally regulated [7]. Calcium binding proteins (Ca^2+^/calmodulin-dependent protein kinase II (CAMKII) and protein kinase C) decode the Ca^2+^ signals to various cellular processes [20,21]. With the completion of the cellular responses, an “OFF” mechanism restores the low concentration of [Ca^2+^]_i_. [Ca^2+^]_i_-signaling is involved in both proliferation and apoptosis. Ca^2+^-oscillations stimulate cell proliferation via Ca^2+^ sensitive transcription factor (NFAT) and conversely, an increase in [Ca^2+^]_i_ for a longer duration activates apoptosis [22]. 

Abnormalities in [Ca^2+^]_i_-signaling are associated with various cancers and is also implicated in therapy resistance [23,24,25]. An extensive review by Cui et al. broadly outlines calcium regulating proteins altered in specific cancer types and enlist those compounds targeting calcium-signaling [7]. In this review we analyze the anti-cancer action of selected agents targeting the calcium dependent pathways regulating proliferation and apoptosis. Here, we emphasize the role of calcium-signaling in proliferation and apoptosis and in addition, highlight calcium dependent modification of tumor energy metabolism and epigenetic modification of genes by anti-cancer agents.

## 2. [Ca^2+^]_i_ -Signaling in Cell Proliferation and Apoptosis

[Ca^2+^]_i_ is a versatile second messenger in both proliferation and cell death. [Ca^2+^]_i_-signaling involves the participation of various proteins combined differently depending upon the type of cellular process initiated (Figure 1). [Ca^2+^]_i_-signaling is spatially and temporally distinct for proliferation or apoptosis [26]. Transition of a normal cell to malignant cell involves altered function, translation, and expression of various proteins involved in the calcium regulation and signaling. Therefore, aberrant regulation of [Ca^2+^]_i_ levels may lead to uncontrolled proliferation and inhibition of apoptosis and thus contribute to carcinogenesis [27]. 

### 2.1. [Ca^2+^]_i_ -Signaling and Cell Proliferation 

[Ca^2+^]_i_-signaling mediated by the channels on the plasma membrane and by exchange of Ca^2+^ between the spatially and temporally separated ER and mitochondria determines the type of down-stream signaling which will be activated. The following section focuses on the association between proliferation and extracellular calcium and the influence of Ca^2+^-channels on proliferation. We will also discuss store-operated calcium entry, the sarco/endoplasmic reticulum calcium ATPase (SERCA), and the ER and mitochondrial axis in proliferation. 

### 2.2. [Ca^2+^]_o_ in Cell Proliferation 

Extracellular calcium ([Ca^2+^]_o_) modulates various cellular processes via calcium channels and extracellular calcium-sensing G-protein coupled receptors, which include calcium-sensing receptor (CaSR) and GPRC6a [21]. Past studies describe [Ca^2+^]_o_ as a key regulator of proliferation in chicken fibroblast [28]. A significant difference in the proliferation rate of normal vs. transformed chicken fibroblast is associated with changes of [Ca^2+^]_o_. Similar observations were made in mouse 3T3 cells, with cell proliferation being dependent on [Ca^2+^]_o_, while a calcium driven mechanism initiated DNA synthesis and cell cycle progression that ultimately resulted in cell division [29,30]. Moreover, the influence of [Ca^2+^]_o_ and its role in proliferation is reviewed in detail by Borowiec [30], emphasizing that [Ca^2+^]_o_ potentially exerts biological actions via sensor proteins on the plasma membrane. CaSR senses [Ca^2+^]_o_ and thus triggers the influx of Ca^2+^ through specific channels and regulates Ca^2+^ absorption and homeostasis in various organs. A reduction of Ca^2+^ influx by blocking calcium channels at the plasma membrane (PM) or reduction of [Ca^2+^]_o_ attenuates cell proliferation [31]. 

### 2.3. Role of Ca^2+^ Channels and Pumps in Proliferation 

**Figure 2:**
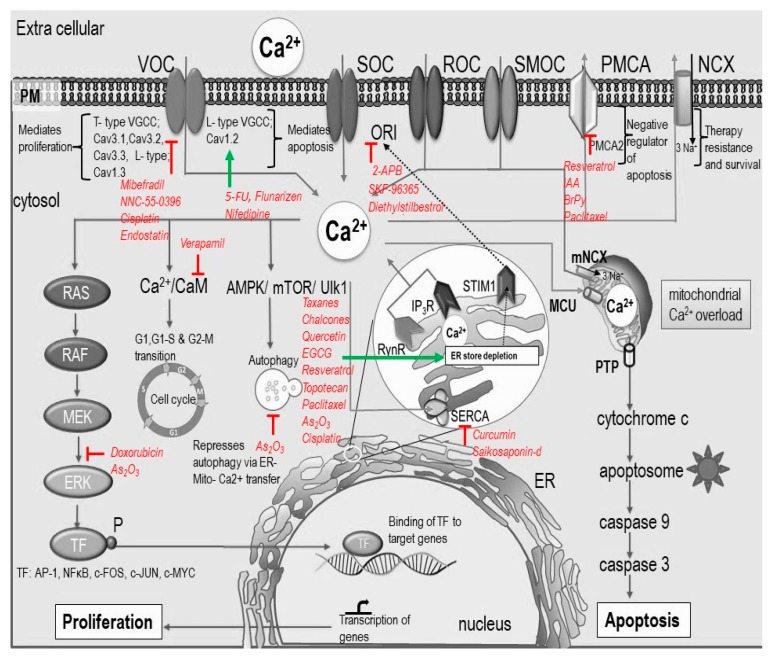
Intra-cellular calcium response to anti-cancer agents. This schematic representation shows the effect of anti-cancer agents on calcium signaling and its downstream effect on apoptosis and proliferation. Anti-cancer drugs can target different calcium channels and pumps on the plasma membrane, ER, and mitochondria. ER-mitochondrial Ca^2+^ transfer plays an important role in apoptosis and many anti-cancer drugs target ER to induce apoptosis. Anti-cancer drugs can also be anti-proliferative (e.g., Doxorubicin) by targeting Ca^2+^-signaling that regulate various proliferation pathways and cell cycle progression. Green arrows represent activation and red lines represent a block.

Ion channels are characterized as protein microchannels regulating intracellular concentrations of ions, contributing to the signaling pathways and influencing the overall behavior of cells [33]. Calcium selective channels are abundantly expressed on the plasma membrane which regulates Ca^2+^-influx. Voltage-gated calcium channels (VGCCs) (also known as voltage operated channels (VOC) (see Figure 1 and Figure 2) sense the depolarization of membrane potentials and open their calcium-selective channel pore, allowing Ca^2+^ entry into the cell, thus contributing to physiological processes [33,34]. Based on their electrophysiological properties, VGCCs are classified into 5 groups: L-type (Ca_v_1), T-type (Ca_v_3), N-type (Ca_v_2), P-type (Ca_v_2), and R-type (Ca_v_2). In non-excitable cells, VGCCs [33] are associated with the regulation of cell proliferation and apoptosis [35]. Aberrant function of VGCCs is related to the progression of different malignancies. Phan et al., in their review, discuss altered VGCC family genes and its link to 19 different cancer types including brain, kidney, breast, and lung cancers [34]. It highlights the downregulation of specific VGCC family-genes in different cancers and emphasizes their role as tumor suppressor genes. Among the different VGCCs, L-type channels show a strong association with proliferation and migration of cancer cells. Moreover, estrogen can modulate the expression of ion channels. A higher expression of L-type channels (Ca_v_1.3) correlates with an increase in 17β-estradiol level in endometrial cancer (EC). The mechanism behind Ca^2+^-induced cell proliferation involves the interaction of estrogen binding to G-protein coupled estrogen receptor (GPER) causing the L-type channel to open and allow Ca^2+^-entry, followed by downstream activation of the ERK1/2-CREB pathway promoting cell proliferation. L-type channels are also involved in migration of EC cells [35,36]. Additionally, a high level of estrogen is associated with increased breast cancer (BC) risk. The mechanism of estrogen-BC risk is linked to a pathway similar to EC proliferation. Importantly, estrogen induced a dose-dependent increase in the expression of Ca_v_1.3 in MCF-7 cells. The silencing of G protein-coupled estrogen receptor 30 (GPER1/GPR30) mediated via siRNA transfection abolished Ca^2+^-influx as well as proliferation in BC, which may be an indication of a crucial role of Ca^2+^ entry through L-type channels in cell proliferation and breast cancer progression [29]. Furthermore, overexpression of the Calcium voltage-gated channel subunit alpha1 D gene (CACNA1D) is associated with prostate cancer progression. Importantly, [Ca^2+^]_i_ measurements showed that an androgen stimulated Ca^2+^ influx was sensitive to nifedipine, indicating that L-type calcium channels were responsible for the androgen-stimulated Ca^2+^-influx in LNCaP cells. Interestingly, blocking of L-type channels resulted in decreased cell growth [37]. 

Similarly, T-type channels are important in various physiological processes like secretion of transmitters and hormones [38]. At the cellular level, these channels regulate cell cycle progression, proliferation, and survival [39,40]. Studies show varying expression of T-type channels in cell cycle phases. A maximum expression (90% increase) of T-type channels is evident in the S-phase and at the beginning of M-phase of the cell cycle. Moreover, increased expression of T-type channels is observed in cancer of breast, bladder, lung, and liver [40]. Knockdown experiments of these channels in MCF-7 breast cancer cells resulted in two different outcomes: The silencing of Ca_v_3.1 enhanced proliferation, and the silencing of Ca_v_3.2 decreased proliferation [41]. As a result, Ca_v_3.1 gene appears to have a tumor suppressor role while Ca_v_3.2 possesses pro survival activity [41]. VGCCs are thus highly expressed in various cancers and thus contribute to the regulation of carcinogenesis. Despite the fact that molecular mechanisms of their activity is still not clear, VGCCs may represent a diagnostic marker and therapeutic target in the cancer treatment [34]. Moreover, PMCA is a major efflux pathway in [Ca^2+^]_i_-regulation, and impaired PMCA function results in Ca^2+^ overload and cell death. Glycolytic inhibition and subsequent ATP depletion impairs PMCA function and induces cells to undergo apoptosis [42].

### 2.4. Store Operated Calcium Entry in Cell Proliferation 

Store operated calcium channels (SOC) on the plasma membrane generate calcium signals in a variety of cell types [43] and are considered to be the main calcium entry in non-excitable cells. Moreover, SOC entry controls a wide range of physiological functions such as apoptosis, proliferation, and migration [44]. Significantly, the activation of SOC entry is mediated via internal Ca^2+^-efflux [8]. Ca^2+^ stored in the ER and the mitochondria actively contributes to [Ca^2+^]_i_ -signals as the Ca^2+^ influx is triggered by a calcium release from the ER which is mediated through stimulation of surface receptors [45]. Two important proteins, STIM1 on the ER (calcium sensor) and Orai1 (pore forming protein) of the calcium release-activated channels (CRAC) on the plasma membrane are involved in SOC entry. Ca^2+^-influx mediated by SOC entry activates “NFAT” transcription factor via STIM1 and Orai1, driving the proliferation pathway [45]. Proliferation is also positively regulated by SOCE (store operated calcium entry) through PI3K/Akt pathway [46]. A dysregulated STIM1/Orai1/SOC axis is implicated in many pathological conditions such as SCID (severe combined immune deficiency syndrome); cardiovascular and pulmonary diseases; and cancer of the breast, colon, and, esophagus [44]. In breast cancer, STIM1 and STIM2 contribute to invasion, migration, and SOC-dependent TGFβ-mediated EMT (epithelial-mesenchymal transition), and their overexpression significantly correlates with the poor survival [47]. Breast cancer is associated with an increased level of Orai1 and knockdown of Orai1 using siRNA in MCF-7 and MDA-MB-231 cells showed reduced SOC activity and decreased number of viable cells [48]. Moreover, in the ER positive BC cell line MCF-7 and T47D knockdown of Orai3 resulted in cell cycle arrest at G1 phase and induction of apoptosis [49]. Furthermore, in colon cancer cells the SOC-dependent migration was mediated by a complex of protein involving SK3/TRPC1/Orai1 [50]. Additionally, inhibition of SOC activity by non-steroidal anti-inflammatory drugs (NSAIDs) attenuated proliferation in the HRT-18 colon cancer cell line [51]. As the SOC entry is considered to be primary Ca^2+^ entry mechanism in most cancer types (thus contributing to cancer cell migration, invasiveness, and metastasis), there is a high interest in development of selective SOC entry blockers to prevent cancer metastasis [52,53]. 

### 2.5. SERCA in Cell Proliferation

SERCA is a calcium pump located in endoplasmic reticulum which maintains a low calcium level and thus enables signaling of various physiological processes [54]. Inhibition of SERCA by thapsigargin empties the ER-Ca^2+^ store and inhibits proliferation. Importantly, different isoforms of SERCA show altered expression in different types of cancers. For instance, SERCA2 is linked to the malignant progression of colorectal SW480 cells while its overexpression is associated with increased proliferation and migration of cancer cells mediated via activation of MAPK and AKT signaling pathways [55]. A close link between SERCA inhibition by thapsigargin and the ER calcium pool content depends on the epidermal growth factor (EGF) concentration, as was observed in LNCaP prostate cancer cells. The results confirmed the increase of ER-Ca^2+^ in the presence of EGF (200 ± 19 μM) when compared to control without EGF (90 ± 5 μM). In addition, higher expression of SERCA2b in the presence of growth factors such as EGF, DHT (dihydrotestosterone) serum, and the ER-Ca^2+^-pool concentration is important in regulating proliferation of LNCaP human prostate cancer cells [56]. A significant difference in [Ca^2+^]_i_ homeostasis is observed in normal human bronchial epithelial (NHBE) cells vs. different lung cancers. Lung cancer cells showed lower ER-Ca^2+^-content which correlated with reduced expression of SERCA2b and increased expression of IP_3_R and calreticulin (a calcium buffering protein in the ER) [57].

### 2.6. ER and Mitochondrial Axis in Proliferation 

High resolution microscopy images show a dynamic network of ER and mitochondria juxtaposed at various domains. These are identified as microdomains with high [Ca^2+^]_i_ related to IP_3_ mediated Ca^2+^-release and are important determinants of [Ca^2+^]_i_-signaling, thus controlling survival, autophagy, apoptosis, metastasis, and invasiveness [58,59]. Mitochondrial calcium homeostasis is essential for cell survival and metabolic regulation [60,61]. Therefore, mitochondria play a dual role in death and survival, and dysregulation of mitochondrial Ca^2+^-homeostasis is associated with various diseases including cancer [62]. Importantly, mitochondrial calcium uniporter (MCU) is the channel enabling accumulation of Ca^2+^ in the matrix [62]. Aberrant expression or functioning of MCU complex contributes to cancer [62]. Significantly, integrity of the mitochondrial membrane, a sustained mitochondrial membrane potential, mitochondrial Ca^2+^ homeostasis regulated by Na^+^/Ca^2+^ exchanger, and MCU may ensure cell survival. Conversely, loss of mitochondrial potential, Ca^2+^ overload and opening of the permeability transition pores (PTP) activates an apoptotic cascade [63]. 

### 2.7. [Ca^2+^]_i_ and Apoptosis 

Programmed cell death, also known as “apoptosis”, is crucial for such normal cell physiological functions as turnover and cell death. There are two main signaling pathways (intrinsic and extrinsic) that culminate in apoptosis [64]. Mitochondria have a dual role in cell death and survival which is well documented. The intrinsic pathway is marked by mitochondrial Ca^2+^ overload, which results in the release of cytochrome *c*. Cytochrome *c* then combines with APAF-1 and, along with ATP and procaspase 9, forms the apoptosome, followed by activation of procaspase 9 to active caspase 9, which triggers the activation of effector caspases downstream, ultimately resulting in the death of the cell. In addition to caspases, calpains are another class of cysteine proteases which require calcium for their activation. Importantly, calpains mediate apoptosis as a response of ER stress [61]. In fact, mitochondria, as the main provider of cellular energy, also function as a regulator of [Ca^2+^]_i_. There is a calcium crosstalk in the domain between ER and mitochondria known as mitochondria-associated membrane (MAM). Importantly, tumor cells modify their MAM which leads to alterations of tumor homeostasis and consequently to promotion of migration, invasiveness, metastasis, resistance to apoptosis, and induction of EMT [63]. Furthermore, [Ca^2+^]_i_ homeostasis also may be influenced by Bcl-2 proteins. Overall, members of the Bcl-2 family play an important role in cell death and survival via modulation of Ca^2+^-transport at the ER, plasma membrane, or mitochondria [65]. 

Furthermore, VDAC1 (voltage-dependent anion channel 1), which is located on the outer mitochondrial membrane (OMM), allows efflux and influx of ions and metabolites. It has a significant role in cell survival and death. It facilitates mitochondrial-mediated cell death in association with apoptotic proteins, causes the release of cytochrome *c*, and induces apoptosis. Furthermore, [Ca^2+^]_i_ rise elevates VDAC1 expression and positively modulate apoptosis [66,67]. SOCE is also involved in apoptosis as SOC channels are activated or opened upon depletion of Ca^2+^ from the ER store. This causes an influx of Ca^2+^ from the extracellular pool, resulting in increased [Ca^2+^]_i_ which is related to activation of apoptosis. The release of Ca^2+^ from ER store as well as influx of Ca^2+^ through the SOC channels influence apoptosis. As reported by Wertz and Dixit, the release of Ca^2+^ from the intracellular store alone activated apoptosis via caspase 3/7 in LNCaP prostate cancer cells [68]. Similarly, Skryma et al. demonstrated no requirement for the Ca^2+^ entry from the store in thapsigargin-treated LNCaP cells [22]. Importantly, the events that contributed to apoptosis following ER calcium depletion led to ER stress and initiation of death signals [69]. In this finding an exclusive relationship between ER-Ca^2+^ store/bcl-2/apoptosis was identified in LNCaP cells treated with thapsigargin [69]. Moreover, inhibition of SERCA by thapsigargin in prostate and breast cancer cells led to drainage of the ER-Ca^2+^ stores, which was followed by reduction in cell proliferation and consequent apoptosis [69].

## 3. Targeting Calcium Signaling for Anti-Cancer Therapy

Anti-cancer drugs have multiple modes of action and, based on their mechanism of action, can interfere at the DNA level, act as antimetabolites, inhibit enzyme synthesis, and inhibit microtubule function. In the past decade, much attention has been drawn to calcium signaling. Altered calcium signaling is crucial for the development and progression of cancer through the induction of proliferation, invasion, and metastasis [70]. Figure 2 and Table 1 summarize anti-cancer drugs and their [Ca^2+^]_i_ mediated induction of cell death.

### 3.1. [Ca^2+^]_o_ Influences Drug Efficiency 

[Ca^2+^]_o_ influences the mechanism of action of paclitaxel in inducing apoptosis in breast cancer cells. Apoptosis induced by low dose paclitaxel is independent of [Ca^2+^]_o_ whereas for high dose paclitaxel, normal [Ca^2+^]_o_ is critical for inducing apoptosis in MDA-MB-468 TNBC cells, which is mediated via capacitative Ca^2+^-entry [81]. Similarly, low [Ca^2+^]_o_ reduced anti-cancer action of tamoxifen (TM) on HepG2, human hepatoblastoma cells. To understand the route of Ca^2+^ entry, Ca^2+^ channel blockers nifedipine and verapamil were used, but neither of them reversed the effect of TM, while flufenamic effectively reversed the effect of TM. A non-selective cation channel (NSCC) blocker caused significant inhibition of TM-induced apoptosis, indicating NSCC as the main route of Ca^2+^-influx [95]. Modifying the [Ca^2+^]_o_ improved the cytotoxic action of CDDP and topotecan [87]. 

### 3.2. Anti-Cancer Drugs-Induced [Ca^2+^]_i_ Modulation Triggers Apoptosis 

#### 3.2.1. Platinum Drugs

Cisplatin (CDDP; *cis-diamminedichloridoplatinum*) is used in treatment of solid tumors and modulates [Ca^2+^]_i_. It blocks voltage gated channels at higher concentrations [96] but allows calcium entry through other channels [31] and releases calcium from the stores [97]. In neuroblastoma (NB) cells, CDDP alters the expression of key calcium regulating proteins such as RyR and IP3R [98]. HeLa cells, when treated with CDDP for 16 h, showed an increase in [Ca^2+^ ]_i_ followed by an increased expression of VDAC1 and oligomerization of VDAC1-triggered cytochrome *c* release and concomitant apoptosis [67]. An increased expression of VDAC1 sensitizes cancer cells to anti-cancer drugs via Ca^2+^-dependent mechanism. A study in HeLa S3 cells revealed a rise in [Ca^2+^]_i_ following CDDP treatment, which correlated with the activation of calpain, an important molecule in the induction of apoptosis [31]. Increase in CDDP concentration caused an increase in [Ca^2+^]_i_ in MCF-7 breast cancer cells [72]. S100A9, a calcium-binding protein, is associated with cancer progression and has been reported to influence squamous cervical cancer cells’ sensitivity to CDDP treatment. Here, down-regulation of S100A9 protein resulted in increased cell death potentiated by altering pro survival AKT/ERK-FOXO1-Nanog signaling pathway [99]. Another study emphasized the role of NCX and NCXK (K^+^ dependent Na^+^/Ca^2+^-exchangers) in cisplatin chemotherapy of ovary carcinoma cells. In this study using A2780 ovarian cancer cell line, the expression of NCX 3 NCKX4, NCKX5, and NCKX6 isoform was higher in the CDDP resistant cell line. NCX inhibitor KB-R7943 (10 μM) significantly improved CDDP sensitivity there by confirming the role of NCX in therapy resistance [100]. Most of the anti-cancer drugs have side effects. Oxaliplatin, a platinum-based drug, undergoes activation with the release of oxalate and the generation of oxalate metabolite is often associated with oxaliplatin associated peripheral neuropathy. Schulze et al. examined the mechanism of oxaliplatin mediated calcium signaling and its role in oxaliplatin-induced peripheral neuropathy. A prolonged exposure to the drug induced changes in the ER calcium load and IP3R mediated calcium signaling, though there was no activation of cellular temperature sensors—TRP channels [101]. Strategies to selectively target cancer cells by sparing the normal cells is a wise approach. The new platinum drug [Pt(O,O′-acac)(*γ-*acac)(DMS)] (PtAcD) is selectively toxic on the breast cancer cell line and less toxic on normal breast cells due to differentially activated JNK and p38 [102]. Research on next generation platinum drugs (e.g., (Pt(IV) carboxylate complexes) are underway with more efficacy and less side effects.

#### 3.2.2. Anti-Metabolites

5-Fluorouracil (5-FU), an anti-metabolite which interferes with DNA synthesis and repair, is used for treatment of breast, skin, pancreatic cancer, etc. Its mechanism of apoptosis induction in HCT116 involved the Ca^2+^-influx from extracellular space, which was detected as early as 1.5 h after exposure to 5-fluorouracil. Consequently, activated p53 was detected after 5 h of 5-FU treatment followed by activation of caspase and induction of apoptosis. Elevation of [Ca^2+^]_i_ is an early event in the apoptosis of HCT116 mediated via Ca^2+^-CaM-p53 axis [74]. Another study reported the involvement of TRPV 1 channel in 5-FU induced cell death in MCF-7 cells, which involved rise in [Ca^2+^]_i_ followed by mitochondrial ROS production and caspase activation [75]. TRPV1 is a non-specific cation channel sensitive to temperature, pH, and capsaicin.

#### 3.2.3. Inorganic Arsenic Compounds 

Arsenic trioxide (As_2_O_3_) is an anti-cancer drug used for the treatment of hematologic cancers such as chronic myelogenous leukemia (CML) [103]. Various studies have reported As_2_O_3_ modulating different Ca^2+^-dependent/independent signal transduction pathways, resulting in the induction of apoptosis, inhibition of angiogenesis, and proliferation [104]. Receptors on the ER (IP3R and ryanodine R) are involved in the induction of apoptosis by elevating [Ca^2+^]_i_. Treatment with 1 µM As_2_O_3_ in SH-SY5Y NB cells elevated [Ca^2+^]_i_ by ER-Ca^2+^ store depletion [24]. Calcium imaging showed three types of signals: A slow and steady increase in [Ca^2+^]_i_, transient calcium elevation, and spikes. Mechanistic studies have revealed increased H_2_O_2_ level, Bax expression, growth arrest at the G1 phase of the cell cycle, and inhibition of vascular endothelial growth factor (VEGF) upon As_2_O_3_ treatment [105,106]. Missiroli et al. [77] elucidated a Ca^2+^-dependent mechanism that coordinately regulated apoptosis and autophagy in cancer. They investigated *pml*, a tumor suppressor gene related to the pathogenesis of acute promyelocytic leukemia. PML protein and its association with ER-mitochondrial contact sites were important in repressing autophagy, and treatment with As_2_O_3_ was shown to reduce the autophagy efficiency by modulating PML protein in NB4 leukemia cells [77]. Similar to CDDP treatment in HeLa cells, increased expression of VDAC1 is observed with As_2_O_3_ treatment in SKOV-3 cells (human ovarian carcinoma) and A549 cells (human lung adenocarcinoma alveolar basal epithelium).

#### 3.2.4. Anthracyclines

Studies on doxorubicin, a chemotherapeutic drug belonging to the category anthracyclines, reported contrasting observations on the effect of Ca^2+^ on doxorubicin cytotoxicity. A counteracting mechanism of Ca^2+^ concentration on doxorubicin activity was reported by Nguyen et al. [107]. Here, [Ca^2+^]_o_ concentration dependently suppressed the cytotoxic activity of doxorubicin in MCF-7 breast cancer cell line. High [Ca^2+^]_o_ (100 and 140 mM CaCl_2_) almost completely rescued the cells treated with 0.2 µg/mL of doxorubicin (LD_50_). Mechanism revealed that Ca^2+^ activated V-ATPase (Vacuolar-type H^+^-ATPase), reduced the bioavailability of drug by acidifying the intracellular organelles and sequestering doxorubicin into subcellular compartments [107]. The finding emphasizes the observation that high [Ca^2+^]_o_ confers insensitivity to doxorubicin treatment. In another study, silencing of PMCA2 calcium channel increases the sensitivity of MDA-MB-231 to doxorubicin treatment. Doxorubicin and simvastatin treatment in breast cancer cells caused a persistent release of Ca^2+^, resulting in activation of proapoptotic BIM pathway and subsequent overload of mitochondrial Ca^2+^ triggering apoptosis. In addition, both drugs significantly reduced the activation of pro survival pathway ERK1/2 signaling, which is calcium-dependent in doxorubicin treatment [79]. 

#### 3.2.5. Taxanes

Paclitaxel is an anti-cancer drug used for the treatment of solid tumors. In neuroblastoma cells (SH-SY5Y), paclitaxel at a low concentration (sub-micromolar concentration) induced Ca^2+^-oscillations independent of [Ca^2+^]_o_ or mitochondrial calcium but dependent on IP3R mediated calcium influx from ER. The molecular basis of paclitaxel induced cytosolic Ca^2+^-oscillation is due to the enhanced binding of protein neuronal Ca^2+^-sensor 1 (NCS-1) to IP3R [108]. Conversely, in MD-MBA-468 breast cancer cells under normal [Ca^2+^]_o_, Ca^2+^ entered from the extracellular pool following low dose paclitaxel (10^−7^ M) treatment and was independent of ER store, but high dose paclitaxel (10^−6^ M) induced Ca^2+^-influx extracellularly and a gradual depletion of ER store occurred, culminating in apoptosis. The mechanism of paclitaxel-mediated apoptosis is dependent on [Ca^2+^]_o_ and dosage of the drug [81]. 

#### 3.2.6. Glucocorticoids

Glucocorticoid (GC), a class of drugs that have multiple physiological effects such as immune suppression, cytotoxicity, and anti-inflammatory effects, is commonly used for hematological malignancies like leukemia, lymphoma, and myeloma [109]. GC modulates [Ca^2+^]_i_ homeostasis in B lymphocytes, while [Ca^2+^]_i_ has a complex role in the induction of apoptosis by glucocorticoids. Release of Ca^2+^ from the stores initiates apoptosis in GC-treated lymphocytes. Here, Ca^2+^-dependent proteases such as calpain were activated, and blocking of calpain activation prevented GC-induced cell death [109]. Similar to doxorubicin, [Ca^2+^]_i_ attenuated dexamethasone sensitivity in acute lymphoblastic leukemia (ALL) cells. Dexamethasone increased [Ca^2+^]_i_ mainly by SOC-operated calcium entry [110]. 

#### 3.2.7. Natural Compounds

Analysis of studies based on some natural compounds showed a Ca^2+^-dependent anti-cancer effect (see Table 1 and Figure 2). For example, Resveratrol induced Ca^2+^-dependent apoptosis through a biphasic [Ca^2+^]_i_-rise involving mitochondria, activation of calpain, and decreased SERCA activity [85,111]. Saikosaponin-d, a natural SERCA inhibitor, induced Ca^2+^ dependent autophagy mediated cell death via CaMKKb-AMPK-mTOR pathway [86]. In another study, epibrassinolide, a polyhydroxylated sterol derivative, induced Ca^2+^ sequestration and thus caused an alteration in the ER pathway, consequent ER stress, and progress to apoptosis [112]. Using a model of endometriosis, Park et al. described that luteolin exerts antiproliferative and apoptotic effects in pre-neoplastic human endometrial cells VK2/E6E7 and End1/E6E7. The anti-cancer effects of luteolin were linked with an increase in cytosolic calcium levels, ROS production, and lipid peroxidation in cells and altered regulation of PI3K/AKT and MAPK cell signaling, as well as the expression of CCNE1 [113]. Using the same model and cells, the same group showed that delphinidin exerts proapoptotic and antiproliferative effects mediated by MAPK and PI3K/AKT signaling proteins. These effects were accompanied by a decrease in the phosphorylation of ERK1/2, AKT, p70S6K, S6 and an increase in the phosphorylation of p38 MAPK and p90RSK [114]. Delphinidin induced apoptosis in human endometrial cells by decreasing the mitochondrial membrane potential and increasing the [Ca^2+^]_i._ Safrole-mediated apoptotic cell death in HSC-3 cells was associated with an increase of cytosolic Ca^2+^ levels, a decrease in the mitochondrial membrane potential, and activation of Fas-dependent pathways [115]. Curcumin induced apoptosis in HepG2 cell, which was associated with the disruption of mitochondrial membrane potential and disturbance of intracellular free Ca^2+^ concentration [116]. Natural products are potential drug candidates which can be considered for combination therapy as this strategy can increase sensitivity to chemotherapy by targeting multiple pathways and, therefore, reduce the side effects. 

#### 3.2.8. Hormonal Receptor Modulator

Tamoxifen (TM) is an estrogen receptor modulator commonly used for breast cancer prevention and treatment. Data from several sources have identified [Ca^2+^]_i_ modulation by tamoxifen, consistent with cytotoxicity in tumor cells [89,91,92]. In ZR-75-1 human breast cancer cells, treatment with tamoxifen at a concentration above 2 μM induced an early [Ca^2+^]_i_ rise due to release from stores as well as entry from extracellular space [91]. A detailed study by Zhang et al. on calcium dynamics in tamoxifen-treated breast cancer cells and glioma cells revealed increase in [Ca^2+^]_i_ level and spatial expansion of calcium waves by 30–150%. Mechanistic studies revealed tamoxifen-induced calcium-depended cytotoxicity facilitated via enhanced purinergic signaling [88]. Furthermore, in human osteosarcoma cells a sustained increase in [Ca^2+^]_i_ due to release of calcium from multiple stores (phospholipase C-independent manner) was recorded following 1 µM tamoxifen treatment [90]. The antiproliferative effect of tamoxifen in human non-melanoma skin cancer cells A431, DJM-1, and HSC-1 is also related to an increase of [Ca^2+^]_i_ [92].

#### 3.2.9. Epigenetic Modulators

Recent studies discovered a prominent role of epigenetic deregulation linked to cancers. The most important epigenetic changes, DNA methylation and histone post-translational modification (methylation and acetylation), result in gene silencing of tumor suppressor genes (TSG) or up/down regulation of other genes. A recent article highlights the role of [Ca^2+^]_i_-signaling in re-activating tumor suppressor genes via CamK. Reactivation of silenced TSG by epigenetic drug azatidine revealed a novel mechanism, which traced back to Ca^2+^-dependent activation of CamK and subsequent release of MeCP2 methyl-binding protein from promotion of the silenced genes [94]. Epigenetic silencing of gene ATP2A3, which codes for SERCA3, is down-regulated in gastric and colon tumors. Treatment of KATO-III cells with butyrate, trichostatin A, and 5-azacytidine increased the expression of SERCA3 and was correlated with increased apoptosis and decreased viability [93]. Mitochondrial Ca^2+^-overload is suggested as a key trigger for programmed cell death. MCU, together with its regulatory subunits, mitochondrial calcium uptake 1 (MICU1) and mitochondrial calcium uniporter regulator 1 (MCUR1), which are responsible for mitochondrial-Ca^2+^ entry, provide novel molecular tools to evaluate this process. Regarding epigenetic modulations of these mechanisms, recent data have also demonstrated that miR-25 reduces mitochondrial Ca^2+^ uptake via MCU, resulting in suppression of apoptosis. It is shown that down-regulation of MCU in human colon cancer cells correlates with miR-25 aberrant expression, pointing the importance of mitochondrial Ca^2+^ regulation in apoptosis.

### 3.3. Calcium Dependent Modulation of Aerobic Glycolysis by Anti-Cancer Agents

Warburg effect (aerobic glycolysis) is a mechanism observed in tumor energy metabolism promoting tumor growth and survival. Here, cells observe excess uptake of glucose and break down of glucose to pyruvate and lactate even in the presence of oxygen and a functional mitochondria [117,118]. Hence cancer cells exhibit altered Ca^2+^-dependent ATPase function. Chakraborty et al. (2017) documented that Mitochondrial Calcium Uptake 1 (MICU1/CBARA1), the gatekeeper of mitochondrial Ca^2+^-uptake, forces aerobic glycolysis and chemoresistance in ovarian cancer cells [119]. Another study showed that an increased expression of transient receptor potential canonical channel (TRPC5) enhances [Ca^2+^]_i_ level and results in chemoresistance and suppressed apoptosis in human colorectal cancer (CRC) cells [120]. In the follow-up of this study, Wang et al. demonstrated the crucial role of glycolysis in TRPC5 induced chemoresistance in CRC cells through maintaining [Ca^2+^]_i_ homeostasis [121]. In vitro studies using dichloroacetate (DCA) demonstrated a shift in the metabolism from glycolysis to glucose oxidation in HeLa cells [122]. The changes in the metabolism modality led to increased intracellular H_2_O_2_ and pH levels, a drop in mitochondrial membrane potential, and the increase of caspase 3 and 9. Regarding mechanism of action, the increased Kv1.5 expression and decreased [Ca^2+^]_i_ appointed a positive feedback loop that caused the decrease in tonic inhibition of caspases. CDDP in combination with DCA exhibited synergy [122]. In vitro studies conducted in human PDAC cell lines (pancreatic cancer) treated with glycolytic inhibitors BrPy 500 μm (3-bromopyruvate) and sodium iodoacetate 2 mm (IAA) resulted in glycolytic inhibition and ATP depletion. As a result, ATP depletion lead to impaired PMCA function and [Ca^2+^]_i_ overload inducing cell death [42] (Figure 2). Furthermore, dexamethasone and prednisolone belonging to the class of glucocorticoids in ALL proved to shift cells’ energy metabolism by suppressing glycolysis and increasing the anti-cancer cancer effect of etoposide [123]. 

### 3.4. Calcium Modulators in Combination with Anti-Cancer Agents 

Tumor heterogeneity can be attributed to the cancer stem cell, tumor microenvironment, gene mutation, and epi-genetic changes. Consequently, cancer cells exposed to chemotherapeutic drugs are often not completely eliminated, and a few cells survive, generating resistant cancer cells. Originally, cancer was treated using single drugs but with the relapse and development of resistance to chemotherapy more trials with drug combinations yielded improved results. Hence, for an effective treatment, the tumor is exposed to a combination of anti-cancer agents targeting different pathways [124]. An important mechanism by which anti-cancer drugs induce cytotoxicity is by interfering with [Ca^2+^]_i_ either by binding or bringing conformational changes to calcium modulating proteins, including channels and pumps located at the plasma membrane, ER, or cytosol [23,25]. A considerable amount of literature has been published on the effect of calcium modulators on cell proliferation and apoptosis [125]. Over the years, research has emphasized the use of calcium modulators for the enhancement of anti-cancer drug efficiency [7]. A report in 1989 claimed a synergistic action of nifedipine with CDDP in B16a-Pt (cisplatin resistant murine tumor cell line) by a mechanism independent of VGCCs [126]. A novel approach using Riluzole, an activator of Ca^2+^-activated K^+^ channel (K_Ca_3.1) and an inhibitor of K_v_11.1 increased the CDDP drug uptake and reversed CDDP resistance in the colorectal cancer cell line [127]. An extensive in vitro study in neuroblastoma chemotherapy using CDDP and topotecan showed enhanced cytotoxic effects with combinations of pharmacological modulators of [Ca^2+^]_i_ regulating proteins [87]. Synergistic action with a panel of calcium channel modulators (cyclosporine A, thapsigargin, dantrolene, 2-APB) in CDDP/topotecan treatment showed promising results in neuroblastoma cell lines SH-SY5Y, NLF and IMR-32 [87]. For example, thapsigargin showed the highest cytotoxic effect by facilitating store-operated calcium entry and triggered apoptosis via ER-mitochondrial axis. Similarly, among the other modulators tested were cyclosporine A and 2-Aminoethoxydiphenyl borate (2-APB), which also induced ER stress and enhanced the cytotoxic effect of both CDDP and topotecan in neuroblastoma cell. 2-APB activates SOC mediated Ca^2+^-entry at a lower concentration, inhibits IP3R, and modulates TRP channels. Live cell calcium imaging studies performed in MCF-7cells pre-treated (30 min) with calcium channel modulators (caffeine, nimodipine, and ionomycin) showed decrease in CDDP-induced [Ca^2+^]_i_ rise [72]. 1,2-Bis(2-aminophenoxy)ethane-*N*,*N*,*N*’,*N*’-tetraacetic acid tetrakis (acetoxymethyl ester) BAPTA-AM is a membrane-permeable calcium chelator widely used for [Ca^2+^ ]_i_-signaling studies_._ A study in HeLa cells (human cervical cancer) indicated that combined treatment with (BAPTA-AM) or 2-APB attenuated CDDP-mediated [Ca^2+^]_i_ rise and apoptosis [31]. The above observations show the role of specific calcium channels in CDDP mediated [Ca^2+^]_i_ rise. As in the case of dexamethasone, BAPTA-AM synergistically enhanced the cytotoxicity of dexamethasone in ALL cell lines [110]. On the contrary, another group reported that doxorubicin efficiency was compromised in the presence of BAPTA-AM in MDA-MB-231 breast cancer cells by preventing the inhibition of ERK1/2 phosphorylation, and was consistent with the attenuation of [Ca^2+^ ]_i_ rise and decrease in cell death [79]. Huang et al. identified a [Ca^2+^]_i_-dependent apoptotic mechanism in PC3 human prostate cancer cells exposed to thapsigargin (1–10 µM). Here, the rise in [Ca^2+^]_i_ was attributed to both Ca^2+^-influx from extracellular environment and depletion of ER stores [128]. Combination treatment with TRAIL (70 and 35 ng/mL) and thapsigargin (0.3 and 0.6 μM) induced apoptosis and inhibited migration, invasion, and adhesion of ESCC cell lines (esophageal squamous cell carcinoma), demonstrating that this combination induces both apoptosis and inhibits metastasis [129]. In MCF-7 and MDA-MB-468 cells, apoptosis was triggered mainly due to a secondary and a delayed (12–36 h) calcium response to thapsigargin (1 µM) [130]. Calcium modulating drugs can be used for enhancement of anti-cancer drug effect, whether it can mitigate side effects of anti-cancer treatment should be explored. Nifedipine, a dihydropyridine-type Ca^2+^-channel blocker improved the blood circulation and reduced hypoxia and hence achieved a better drug delivery towards tumor in an isolated limb perfusion experiment performed in rat bearing tumors [131]. Similarly, verapamil, a calcium channel blocker, improved the sensitivity of tumor to radiation by modifying the tumor vasculature [132].

Collectively, the combination of calcium channel modulators with anti-cancer drugs can have a beneficial effect with some combination of drugs.

## 4. Conclusions and Future Directions 

Cancer research reveals that [Ca^2+^]_i_-signaling has substantial effects on proliferation and apoptosis. Prolonged increase in [Ca^2+^]_i_ triggers apoptotic cascade and is the principle behind the action of many anti-cancer drugs. In the context of cancer, many drugs such as CDDP, topotecan, and As_2_O_3_ induce apoptosis depending on extra- and intracellular calcium. The modification of either [Ca^2+^]_o_ or [Ca^2+^]_i_ by calcium chelating agents can significantly augment the action of anti-cancer drugs such as doxorubicin and dexamethasone. This observation is relevant in a clinical context as caution should be exercised when using calcium-altering drugs and diets. Calcium signaling plays a dual role in cell survival and death. ER–mitochondrial Ca^2+^ fluxes are crucial in steps (cancer hallmarks) leading to cancer growth, and it is the most targeted site for many of the anti-cancer drugs (Figure 2). ER-mitochondrial Ca^2+^-flux, SOC entry, channels, and pumps on the PM coordinately control the fate of the cell. Hence, components of the calcium tool kit are potential targets of anti-cancer drugs. Nuclear calcium controls gene regulation, but its dependence on cytosolic calcium is a subject of debate. Hence, a drug which modulates [Ca^2+^]_i_ may or may not affect nuclear calcium, but there is no adequate information on this. 

Dysregulated expression of many calcium regulation proteins is associated with migration, proliferation, apoptosis, angiogenesis, and invasion in cancer. However, it is beyond the scope of this review to discuss all the calcium signaling components dysregulated in various cancers. A growing body of evidence emphasizes targeting altered calcium homeostasis in cancer as a potential tool in cancer chemotherapy by modulating either the expression of calcium regulating proteins or their function. CDDP and topotecan are drugs identified to modulate the expression of IP_3_R and ryanodine in cancer chemotherapy, however, there are multiple calcium channel inhibitors/activators, which block/activate the function of various channels and thus modulate the apoptosis and proliferation of the cells. In this regard, many combinations of classical drugs with calcium channel regulators have improved efficacy within cancer chemotherapy. Moreover, a wide variety of drugs derived from natural compounds have proapoptotic and antiproliferative modes of action, such as topotecan, anthracyclins, and taxanes, and all modulate calcium signaling. Curcumin, safrole, luteolin, delphinidin, and other phytochemicals can potentially serve as molecules for developing novel anti-cancer drugs targeting proliferation and apoptosis in cancer cell via regulation of cytosolic Ca^2+^-levels. Reversing the glycolytic phenotype may trigger apoptosis in tumor cells, and thus represents an attractive therapeutic tool for anti-cancer clinical strategies. Selective targeting of the key functional enzymes of glucose metabolism through the elevation of [Ca^2+^]_i_ level, which could lead to [Ca^2+^]_i_ overload and promote apoptosis, is a new challenge for oncological research. Targeting epigenetic pathways is a newer approach in cancer chemotherapy, and using the calcium signaling pathway to reactivate silenced genes was recently introduced. In this regard, certain calcium-modulating agents have been proven to epigenetically increase the protein phosphorylation included in apoptosis/cell-cycle signaling, which is repressed in several cancers such as in the liver, pancreatic, breast, or prostate.

An increasing number of studies have identified and listed various proteins in the calcium signaling tool kit which are altered in various cancers [1]. Studies emphasize the important role of calcium cell signaling and various calcium regulating proteins in cancer development. However, further investigations are needed to explore precise molecular mechanisms of their anti-cancer action. Developing drugs against targets involving the calcium signaling tool kit is challenging as these proteins are ubiquitously present in all types of cells. It is important to critically classify those targets that are relevant to each type of cancer.

## Figures and Tables

**Table 1:** Interference of anti-cancer agents with different mechanisms of calcium homeostasis. This table illustrates which calcium mediating processes are involved, the actual concentrations (doses) needed, the experimental model, and whether apoptosis or proliferations are involved.

Drug Class	Drugs	Axis/Mechanism of Induction of Cell Death	Concentration Range	Apoptosis	Proliferation	Cell Line	References
Platinum agents (cytotoxic alkylating agent)	*Cisplatin*	[Ca^2+^]_i_ ↑↑ by influx of extra cellular calcium. [Ca^2+^]_i_ ↑↑ / ER stress / mitochondrial Ca^2+^ over load / caspase 3 activation.	1 µM 5 µg/ml	↑↑	↓↓	MCF-7 SH-SY5Y HeLa-S3	[31,71,72,73]
Anti-metabolites	*5-Fluorouracil*	Ca^2+^-CaM-p53 activation, Ca^2+^ influx partially through L-type Ca^2+^ channel. Ca^2+^ entry through TRPV1 / mitochondrial ROS production / caspase 8.	768 μM, 25 μM	↑↑	↓↓	HCT116 MCF-7	[74,75]
Inorganic arsenic compounds	*As_2_O_3_*	IP3R, RyR / [Ca^2+^]_i_ ↑↑ / DNA damage / caspase 3. ↑↑ER–mitochondrial Ca^2+^ transfer. ↓↓ ERK1 and ERK2	1 µM	↑↑	↓↓	SH-SY5Y Pml*^-^*/*^-^* mice NB4 cells U937	[31,76,77,78]
Anthracyclines	*Doxorubicin*	[Ca^2+^]_i_ modulation - ERK1/2 inactivation, activation of pro apoptotic BIM pathway and mitochondrial Ca^2+^ overload.	500 nM–1 µM	↑↑	↓↓	MDA-MB-231	[79]
Taxanes	*Paclitaxel* *Docetaxel*	In activation of PMCA2/calcineurin A and activation of calcineurin A /NFAT pathway/ ↑↑ pro-apoptotic protein Fas ligand. External calcium influx, inhibition of bcl2/ IP3R-ER-[Ca^2+^]_i._	1 nM 10^-6^ M	↑↑		MDA-MB-231 MCF-7 MDA-MB-468	[80,81]
*Natural compounds*	*Chalcones* *Quercetin* *EGCG* *Piceatannol Etoposide; (semi-synthetic)* *Resveratrol* *Curcumin* *Saikosaponin-d*	Ca ^2+^ / ER stress / caspase 12. G-protein / IP3R-ER-[Ca^2+^]_i,_ / modulation of p53 / transcription of pro-apoptotic genes. SERCA↓↓ activity / [Ca^2+^]_i_ ↑↑ / increased mitochondrial Ca^2+^ uptake. SERCA ↓↓ / [Ca^2+^]_i_ ↑↑ / ER stress / Autophagy mediated cell death.	30–40 µM 50–100 µM 10 µM	↑↑		L1210 MDA-MB-231 MYCN2 HeLa SW480 (colon) MCF-7	[55,82,83,84,85,86]
*Camptothecin analog*	*Topotecan*	Increased [Ca^2+^]_i,_ altered expression of calcium regulating proteins.	0.01 µM	↑↑	↓↓	SH-SY5Y	[87]
*Hormonal receptor modulator*	*Tamoxifen*	[Ca^2+^]_i_ ↑↑ by influx of extra cellular calcium and release of Ca^2+^ from multiple stores. VGCC	5–10 µM	↑↑		MCF-7 MG63 ZR-75-1 SCC BFTC	[88,89,90,91,92]
DNA methylation and HDAC modulators	*TSA* *Azacitidine* *Digitoxin* *Pyrithion zinc* *Disulfiram*	↑↑SERCA3 / apoptosis. SOC / [Ca^2+^]_i_ ↑↑ / CamK / via MeCP2 / reactivation of tumor suppressor genes.	50 nM–5 µM	↑↑		KATO-III (gastric carcinoma) YB5 (colon)	[93,94]

## Abbreviations

[Ca^2+^]_i_	intracellular calcium
[Ca^2+^]_o_	extra cellular calcium
ALL	acute lymphoblastic leukemia
APAF-1	apoptotic protease activating factor 1
As_2_O_3_	arsenic trioxide
ATP	adenosine triphosphate
BrPy	3-bromopyruvate
Calreticulin	calcium buffering protein in the ER
CamK	Ca^2+^/calmodulin-dependent protein kinase
CaSR	calcium sensing receptor
CDDP	cis-diamminedichloridoplatinum(II)
CML	chronic myelogenous leukemia
CRAC	channel Calcium release-activated channels
CREB	cAMP response element-binding protein
DJM-1	a human skin squamous carcinoma cell line
EC	endometrial cancer
EGF	epidermal growth factor
EGCG	Epigallocatechin gallate
EMT	epithelial–mesenchymal transition
ER	endoplasmic reticulum
ERK1/2	extracellular signal–regulated kinases
GC	glucocorticoid
GPER	G protein-coupled estrogen receptor
HSC-1	a human skin squamous carcinoma cell line
IAA	sodium iodoacetate
IP3R	inositol trisphosphate receptor
IAA	sodium iodoacetate
MAPK	mitogen-activated protein kinase
MCU	mitochondrial calcium uniporter
mTOR	mechanistic target of rapamycin
NB	neuroblastoma
NCS-1	neuronal Ca^2+^ sensor 1
NHBE	normal human bronchial epithelial
NSAID	non-steroidal anti-inflammatory drugs
Orai1	calcium release-activated calcium channel protein 1
PMCA	plasma membrane calcium ATPase
PML	promyelocytic leukemia protein
PTP	permeability transition pore
RyR	ryanodine receptors
SCID	severe combined immune deficiency syndrome
SERCA	sarco/endoplasmic reticulum Ca^2+^-ATPase
SK3	small-conductance calcium-activated potassium channel
SOC	store operated channel
STIM1	stromal interaction molecule 1
TGFβ	transforming growth factor beta
TM	tamoxifen
TF	transcription factor
TSA	trichostatin A
TRPC1	transient receptor potential channel 1
TRPV1	transient receptor potential vanilloid 1
V-ATPase	vacuolar-type H^+^ -ATPase
VDAC1	voltage-dependent anion channel
VGCC	voltage gated calcium channel
VGEF	vascular endothelial growth factor
VOC	voltage operated calcium channel
2-APB	2-Aminoethoxydiphenyl borate
5-FU	5-Fluorouracil
